# Supplementary material for: Effects of image homogeneity on stenosis visualization at 7 T in a coronary artery phantom study: With and without B1-shimming and parallel transmission
Source: PLoS One. 2022 Jun 29;17(6):e0270689. doi: 10.1371/journal.pone.0270689 (PMC9242506; doi:10.1371/journal.pone.0270689)
Supplement: Attachment — Submitted filename: Response to reviewers.docx [file pone.0270689.s002.docx]

Response to Reviewers

Manuscript ID: PONE-D-21-01916

Editor: It is highly recommended therefore to address the methodological concerns raised by the reviewers. In addition whilst we do not judge manuscripts for perceived novelty, it is recommended to address the reviewer's concerns regarding the rationale for experimental choices and the context for this work.

Reviewer Comments to the Authors

Reviewer 1:

In this article, the authors investigated the effects of B1-shimming pTX on image homogeneity compared with pTX without B1-shimming on ultra-high field cardiac magnetic resonance imaging, they compared the measurements of stenosis using phantoms filled with gadolinium-based contrast agent (for Gd-DOTA) performed on images acquired with B1-shimming pTX with those obtained from images acquired with commercial sTX, they rated qualitatively the subjective evaluation of the stenosis for different Gd-DOTA dilution series, and they compared SNR measurements on images acquired with B1-shimming pTX compared with sTX. I believe this study is interesting to the field and has been performed elegantly and properly. The research objectives are well-defined, and the study design is appropriate. The figures and tables summarizing the main results are good. The article is well-written and its structure and organization are also good. The discussion section is informative and are contextualized with the results from peer-reviewed articles relevant to the topic. The references are in general appropriate, and include both newer and older studies. The conclusions drawn by the authors are in general supported by the data they present.
I have only a few minor points:

Comment 1:

The authors should clarify how many measurements per type of stenosis have been performed by reader 2.

***Response:***

*We thank the reviewer for this comment. Reader 2 performed 16 measurements per type of stenosis. We have clarified this in the results and figure legends sections.*

Comment 2:

Similarly, in figure 6, it will probably be more informative to show all the data points in addition to the mean and standard deviations of the bar plots.

***Response:***

*We thank the reviewer for this comment. Fig. 6 is meant to show the measurement accuracy in stenosis quantification using sTX and pTX coil with B_1_-shimming:*

*Fig. 6*

*Additional corresponding graphs showing all data points for sTX and pTX are depicted below:*

*PTX (n=15 per type of stenosis)*

*STX (n=15 per type of stenosis)*

*The authors believe that the inclusion of these graphs would not improve clarity and readability of the manuscript. Therefore, we would like to omit the additional graphics, provided that the reviewer and the editor agree.*

Comment 3:

“Although RF shimming is a promising technique, it also has its limitations [36].” It would be interesting to list a few of them.

***Response:***

*We thank the reviewer for pointing out this important aspect. A corresponding paragraph was added to the discussion section.*

*“In particular, it requires a robust measurement of B_1_-field maps of individual transmit channels. Currently, the vendor-integrated B_1_-calibration procedure takes about 40 sec in non-triggered mode, but elongates to 2 minutes by activation of cardiac gating, which would be a requirement for accurate B­_1_-field maps. This is not compatible with breath-hold measurements and research developments of calibration methods was required.^37^ Moreover, currently, there is no vendor-provided subject-specific SAR safety concept available for the application of pTX-based B­-shimming (both static and dynamic) for thorax pTX-arrays in human subjects. Only a so-called pTX-compatibility-mode can be used with commercially available arrays. Here, B_1_-shimming settings are computed for generic electromagnetic body models („Duke“, „Ella“ etc.) and fixed for a specific coil. Operating with B_1_^+^-shimming in 7T thorax MRI, in general, requires the availability of highly experienced personnel and infrastructure for the electromagnetic simulations ensuring the SAR safety.”*

*[37] Aigner, C.S., Dietrich, S., Schmitter, S., 2021. Three‐dimensional static and dynamic parallel transmission of the human heart at 7 T. NMR in Biomedicine 34.. doi:10.1002/nbm.4450*

Reviewer 2

In this study, the authors investigate the impact of B1-shimming on image homogeneity and stenosis evaluation at 7T in a phantom filled with with gadolinium based contrast agent. They also compare these results with those obtained with a single channel transmit coil.
While in principle the long term target is relevant in terms of public health (coronary stenosis evaluation with MRI at 7T), this study unfortunately does not bring new knowledge, and suffers from serious methodological shortcomings preventing the work from having meaningful relevance to real in-vivo situation. Here are a few examples of such deficiencies:

Comment 1:
The authors chose a static phantom, whereas blood flow is a fundamental and critical characteristic of MR angiography.

***Response:***

*We agree with the reviewer’s valuable point. This is a first pilot study focusing on the effects of image homogeneity on stenosis visualization in a coronary artery phantom study, where the main aim was to assess capability and limitation of the vendor-provided shimming platform. Unfortunately, the current experimental setup does not allow the assessment of flow characteristics and motion of the heart through the cardiac cycle. However this is an important aspect and may be subject to future experimental studies. We further comment on limitations regarding phantom in other responses below.*

*The following paragraph was added to the discussion:*

*“The phantom setup used in this study is relatively simple and direct translation of observed B_1_-inhomogeneity to the human body is limited. However, at 7T the B_1_-profiles in the thorax vary significantly with the subject weight, thorax shape, body mass index and fat-muscle distribution and no single phantom however complex could accurately represent this variety. We believe that the rather simple setup can already provide B_1_-conditions relevant for imaging of vascular stenosis, in particular considering the aim of this study: to assess capability and limitation of the vendor-provided shimming platform using a pTX-array. In addition to cardiac MRI the coils used in this study may be applicable to imaging of the lower extremities. Thus, results of this study may be applicable to such imaging applications as well, indicating that the vendor-integrated shimming process may already improve imaging conditions with respect to B_1_-inhomogeneity and therefore, also improve stenosis visualization in vessels of lower extremities. “*

Comment 2:

Multiple studies have carefully described ways to address the very challenging B0 and B1 inhomogeneities occurring in the torso at 7T. Here, the chosen phantom is not representative of real torso anatomy, whereas it has been described elsewhere, and in details, how B1 shimming and B0 shimming solutions need to be specifically optimized to their target in cardiovascular imaging at 7 Tesla.

***Response:***

*Thank you for this comment. The phantom was filled with a liquid featuring dielectric properties of muscle tissue as described in the literature by Duan et al. for high-field magnetic resonance imaging applications. In our experience, considering the overall complexity of the electromagnetic energy distribution patterns in the body at UHF MRI, it is practically impossible to imitate it just mimicking “an average” thorax shape without reproducing the inner thorax structures (lungs, hear, liver, …). Even a “global” B_1_-profile in the thorax varies very significantly with the subject weight, thorax shape, body mass index and fat-muscle distribution as we have found in our recent study with about 80 volunteers**^1^**. Thus, there is very limited gain in creating more complex phantoms trying to reproduce realistic *local* B_1_-conditions, which anyway would be practically random in terms of distribution of positive and negative interferences, considering a real human subjects cohort. Thus we believe that a basic phantom can also potentially produce *local* B_1_-conditions relevant for imaging of vascular stenosis similar to the one which should be managed by B_1_-shimming in a real subject. Moreover, if we include the possibility of angiography of other body areas (e.g femoral arteries branches) the exact imitation of surrounding by the phantom would be even less reasonable considering the goal of trying to challenge and test the B_1_-shimming capabilities in different conditions. We have added this information in the limitations section.*

Comment 3:

The "non-B1 shimmed" setting taken as a comparison basis here seems fairly meaningless: it is very well known that at 7T B1 shimming are needed to address B1 inhomogeneities and several approaches have been proposed for these challenges. Here, the authors simply rely on the vendor-based B1 mapping and shimming technique without reporting any details on parametric quantities. The "non B1 shimmed" setting seems to be defined arbitrarily at best. RF efficiency and related SAR impact are not discussed. All these considerations can be critical for a technique meant to be ultimately applied in humans.

***Response:***

*We agree that “non-B_1_-shimmed” images are expected to be sub-optimal and this was one of the goals of the study: to challenge the vendor B_1_-shimming platform and the pTX array prototype (nowadays available as a product) to resolve the sub-optimal B_1_ conditions. We updated the introduction to make it more clear for the audience. We believe that in this case it’s quite reasonable to compare images before/after shimming to demonstrate the B_1_-shimming effect. The RF efficiency of the array and SAR impact are of course very important to consider in B_1_-shimming adjustments. This work, however, was primarily focused on the capability of the available pTX platform to address the B_1_-inhomogeneities on the scale practically relevant for MR-imaging a vascular stenosis. The compatibility of the obtained B_1_-shimming settings with the SAR limitations should be based on a specific SAR safety concept for pTX arrays (e.g VOP-matrices) and performing EM-simulations with different human voxel models. This is a subject of current and future work, but was beyond of the scope of this specific study.*

Comment 4:

This study does not include electromagnetic simulations that have become almost unavoidable when addressing transmit B1 issues with multiple channels at 7T.

***Response:***

*We agree that as the next step such simulations should be done. Moreover, for the used transceiver array prototype we have already performed quite extensive electromagnetic simulations in order to optimizing B_1_-conditions for different patient cohorts using both “phase only” adjustments for 16Tx elements and static pTX adjustments with RFPA in pTX-compatibility mode. Results were presented in an ISMRM abstract:*

*[Customized B1+-Shaping using Multi-Channel Transceiver Array Prototype for 7T Cardiac MRI with Central Elements Symmetry](https://cds.ismrm.org/protected/20MPresentations/abstracts/0747.html). Maxim Terekhov, Ibrahim A. Elabyad, Frank Resmer, Titus Lanz, Theresa Reiter, David Lohr, Wiebke Schlöttelburg, and Laura M. Schreiber. Proceedings of the 29th annual meeting of the ISMRM. 2020*

*However, for this specific work one cannot use available human body voxel models such as “Ella” and “Duke”, because they do not present a blood vessel with the resolution suitable for the simulations of the stenosis. The dedicated high-resolution electromagnetic model of the specific vessels branches should be designed to make such simulations practically reasonable. For example, the models used for CFD simulations of the blood flow may potentially be used in the future for this purpose.**^2^* *However, again, this is quite extensive work (including the generation of vascular models suitable for EM-simulations) that would be a subject of an independent paper.*

Comment 5:

There is no description of the single transmit RF coil (not even the size is reported)

***Response:*** *We added a description of housing dimensions for both coils to the method section.*

*The following text was added:*

*“Width, length and height of the anterior and posterior housings of this array are 390mm, 350mm, and 80mm as well as 580mm, 550mm, and 50mm, respectively.”*

*…*

*“Width, length and height of the anterior and posterior housings of this array are 346mm, 330mm, and 90mm as well as 345mm, 455mm, and 48mm, respectively. Both coils are depicted in Fig.1.”*

Comment 6:
Even before considering the aforementioned problems, the purpose of the study seems quite elusive from the beginning. Indeed, in experiments like the ones reported by the authors, it is perfectly known that poor B1 profiles will occur (non shimmed multi channel transmit coils at 7T) and that the latter will translate in very poor and heterogeneous Signal to Noise Ratio (SNR) (multiple studies have reported this in details). Observing poorer precision in diameter or stenosis evaluation when SNR is low and heterogeneous is a straightforward and fully expected consequence. That transmit B1 contributes to this deleterious effect is as much predictable. Likewise, the comparison between the impact of single or multi channel transmit coil does not tell more than what is already known about transmit B1 characteristics.
As a result, this reviewer could not identity relevant conclusions coming from the study.

***Response:***

*The authors agree with the reviewer that the problem of B_1_-induced artifacts and as a consequence degradation of SNR leading to the problems of extracting quantitative information from the images could probably be expected a priory based on previously published 7T MRI studies. However, we see several reasons why the results of the dedicated study on this subject is of interest for the audience:*

1) *7T whole-body MRI is still very much a research field and the specific impact of the above-mentioned B_1_-artefacts for the great number of specific medical imaging aims (e.g. quantification of vascular stenosis) are mostly unknown to non-MR-physicists, i.e radiologists, MR-cardiologists, and other medical specialists using MR-imaging both in daily routine and research. To our knowledge, the problem covered in this work has not been addressed specifically in previous publications on UHF MRI.*
2) *Most of the UHF MRI studies in human subjects (and all of them using pTX technique and pTX-RF-coils) do require ethics approval from the relevant authorities. To justify a human study, a preliminary technical information regarding benefits and drawback of 7T MRI for a specific problem is desirable. The same is valid for studies using large animals (Animal Care Application is necessary).*
3) *pTX-arrays do not belong to CE/FDA approved hardware (even if provided on a commercial basis by the few vendors worldwide). Using these arrays in humans requires involving highly qualified personnel, expensive professional software for EM-simulations and computational resources to provide SAR-safety analysis. Before investing such resources in a project addressing any specific problem, a preliminary study using a phantom is highly desirable.*

*Therefore, we consider this phantom study a necessary preliminary step that should provide information on what could be potentially achieved with both types of arrays (sTX and pTX). Such information may also motivate further studies on pTX-technology (some of which have been discussed in this revision) and potential improvements for the visualization and quantification of vascular stenosis. Accordingly, we have added more information in the introduction setting.*

Comment 7:

Page 4, line 7. MRI is not a radiation-free imaging technique as claimed by the authors. It is free of ionizing radiation, but electromagnetic waves are radiations, even at radio frequencies. This language needs to be modified.

***Response:***

*We thank the reviewer for this comment. The mentioned formulation has been adapted as recommended.*

*Literature:*

***1.***	*Reiter T, Lohr D, Hock M, Ankenbrand MJ, Stefanescu MR, et al. On the way to routine cardiac MRI at 7 Tesla - a pilot study on consecutive 84 examinations. PloS one. 2021;16(7):e0252797. DOI: 10.1371/journal.pone.0252797*

***2.***	*Martens J, Panzer S, van den Wijngaard J, Siebes M, Schreiber LM. Influence of contrast agent dispersion on bolus-based MRI myocardial perfusion measurements: A computational fluid dynamics study. Magnetic resonance in medicine. 2020;84(1):467-483. DOI: https://doi.org/10.1002/mrm.28125*
