# Supplementary material for: Effects of image homogeneity on stenosis visualization at 7 T in a coronary artery phantom study: With and without B1-shimming and parallel transmission
Source: PLoS One. 2022 Jun 29;17(6):e0270689. doi: 10.1371/journal.pone.0270689 (PMC9242506; doi:10.1371/journal.pone.0270689)
Supplement: Attachment — Submitted filename: Response to reviewers.docx [file pone.0270689.s003.docx]

Response to Reviewers

Manuscript ID: PONE-D-21-01916R1

Reviewer Comments to the Authors

Reviewer 1:

I appreciate the authors adding the graphs with all data points in figure 6, which add transparency to the actual analysis performed. I believe these additional graphs are important and should be included in figure 6. However, I have several concerns related to these graphs and in general to figure 6:

Comment 1:

Please make sure that all data points are visible in the additional graphs on panel b and c: there might be some overlapping points which prevent them to be visible (see for example for stenosis type 0%, only 1 point is visible, both in panel b and in panel c, instead of the stated 15 points).

***Response:***

*We thank the reviewer for this comment. We have revised figure 6 with particular emphasis on better visibility. Panels “b” and “c” have been reworked and combined to new figure 6 part 2. To visualize the individual data points, the values on the x-axis (true degree of stenosis) are not fixed to a single value (e.g. 20%), but have a range of +/-5% (e.g. 15% to 25%). The figure legend has been adapted as well.*

*New fig. 6.*

Comment 2:

The y-axis name “mean absolute difference” in panels b and c is possibly incorrect, since negative values are also present.

***Response:***

*The authors agree with the reviewer that the y-axis name “mean absolute difference” was incorrect. We have now combined information from prior figure 6 “panel b and c” into one figure (Figure 6 part 2) and have changed the y-axis name accordingly.*

Comment 3:

The legend for panel a currently reads “Error bars indicate the range of the absolute differences across the phantoms”, which seems incorrect since, also in this case, the error bars include negative values.

***Response:***

*Thank you for this comment. The legend for figure 6 part 1 (“panel a”) has been changed from “absolute differences” to “mean differences”.*

Comment 4:

The error bars in panel a are confusing because they look to be the opposite of the actual data shown in panels b and c: if I understand this correctly, the top of the error bar represents more negative differences, whereas the bottom of the error bar represents more positive differences. My recommendation is to report the mean difference in panel a, rather than the mean absolute difference. This would also facilitate the interpretation, showing that there is overestimation of the MRI measurement compared with real measurement rather than underestimation (which might not be obvious to all the readers). If the authors want to follow this recommendation, please edit the y-axis and the text, and in the figure legend please specify whether the difference is “MRI – real” or “real – MRI”.
If the authors want to keep the mean absolute difference, please specify in the figure legend the sign of the error bars.

***Response:***

*The reviewer is right in his impression that the data in “panel a” looked to be the opposite of the actual data in “panel b and c”. Actually, in our rebuttal letter the data in “panel b and c” were flipped. In fact, mean differences were obtained by subtracting real measures of the 3D-printed phantoms from the MRI measurements. We apologize for this mistake and have corrected the graph. Figure 6 does now include a new part 2, which contains data from “panels b and c” as described above. Furthermore this point has been clarified in the figure legend.*

Comment 5:

In the text, the authors wrote: “Quantification of stenoses using the sTX coil slightly overestimated high-grade stenoses by up to 6.1±1.8%”. Therefore, I suppose that the differences (not absolute) in panels b and c are obtained by subtracting the real measure from the MRI measurement. Please clarify this point in the legend of figure 6 panels b and c.

***Response:***

*The reviewer is right in pointing out this aspect. It has been specified in the legend of figure 6 as recommended.*

Reviewer 2

Comment 1:
PDF page 4, before-last line, typo. The last sentence on this page starts with "1However..." instead of "However..."

***Response:***

*We thank the reviewer for this hint and have changed the typo accordingly.*
